# Editorial: Targeting cancer-associated fibroblasts: disrupting immune evasion and therapy resistance

**DOI:** 10.3389/fimmu.2025.1728782

**Published:** 2025-11-17

**Authors:** Ting Wang, Zhaokai Zhou, Qiong Lu

**Affiliations:** 1 Department of Cardiopulmonary Rehabilitation, Nanjing Zijin Hospital, Nanjing, China; 2 Department of Urology, The Second Xiangya Hospital of Central South University, Changsha, China; 3 Department of Pharmacy, The Second Xiangya Hospital, Central South University, Changsha, Hunan, China

**Keywords:** cancer-associate fibroblasts, immune evasion, therapy resistance, tumor micro environment (TME), extracellular matrix, targeted therapy

The tumor microenvironment (TME) is a complex ecosystem that plays a critical role in cancer progression and therapeutic response (1). Within this network, cancer-associated fibroblasts (CAFs) have emerged as master regulators, and they critically shape an immunosuppressive and therapy-resistant niche in TME (2). In the past, CAFs were merely considered as static structural components. But now, CAFs have been recognized as functionally heterogeneous and metabolically active players that promote tumorigenesis through diverse mechanisms such as extracellular matrix (ECM) remodeling, paracrine signaling, and metabolic reprogramming (3). Their profound impact on anti-tumor immunity and chemotherapeutic efficacy makes them as a promising therapeutic target for overcoming treatment failure in multiple cancer types.

This Research Topic, *“Targeting Cancer-Associated Fibroblasts: Disrupting Immune Evasion and Therapy Resistance,”* was established to compile cutting-edge research and insightful reviews that elucidate the multifaceted roles of CAFs and explore innovative strategies to disrupt the pro-tumorigenic functions of CAFs in different cancer types including esophageal squamous cell carcinoma (ESCC), lung cancer, osteosarcoma, hepatocellular carcinoma (HCC), clear cell renal cell carcinoma (ccRCC), and others—and highlight promising strategies to target these stromal players in cancer therapy.

This Research Topic comprises nine articles, including two original research articles and seven reviews, which systematically elucidate the biology of CAFs across various malignancies. Gao et al. demonstrated that high expression of APE1 in ESCC is associated with increased infiltration of FOXP3+ regulatory T cells (Tregs) and CAFs, correlating with poor survival following adjuvant chemotherapy. This study not only identifies APE1 as a prognostic biomarker but also links it to CAF-mediated immunosuppression, suggesting that stromal-immune crosstalk is a key mechanism of chemoresistance. Similarly, the influence of the stromal environment on tumor behavior is evident in the work of Maeda et al., who showed that an interstitial pneumonia (IP) lung microenvironment promotes metastasis to mediastinal lymph nodes and contralateral lungs in a murine model. This pro-metastatic niche was mitigated by pirfenidone, an anti-fibrotic agent, pointing to the therapeutic potential of modulating the stromal compartment to impede cancer progression. The immunosuppressive functions of CAFs are mediated through diverse mechanisms, including cytokine secretion, metabolic reprogramming, extracellular matrix (ECM) remodeling, and exosome-mediated communication. Luo et al. and Wang et al. elaborate on how CAFs engage in dynamic crosstalk with immune cells—such as T cells, macrophages, and Tregs—to establish an immune-excluded or immune-suppressed TME. These interactions often involve key signaling pathways like TGF-β, IL-6/STAT3, and CXCR4, which not only support tumor survival but also blunt the efficacy of immune checkpoint inhibitors (ICIs). In hepatocellular carcinoma, Shi et al. describe the cellular origins and functional heterogeneity of CAFs, emphasizing their role in immune evasion and chemoresistance. The complexity of CAF subsets—each with distinct marker expression and functional profiles—complicates therapeutic targeting but also offers opportunities for precision medicine. A common theme across these studies is the role of fibroblast activation protein (FAP), a surface protease highly expressed on a subset of CAFs. Lee et al. and Wang et al. discuss FAP as a promising therapeutic target. FAP-positive CAFs contribute to ECM remodeling, angiogenesis, and immune suppression, making them attractive for interventions such as FAP-targeted CAR-T cells, antibody-drug conjugates, or small-molecule inhibitors. However, the heterogeneity of CAF populations and the lack of specific biomarkers remain significant challenges for clinical translation. In addition, nanotechnology offers a promising avenue to overcome these hurdles. Xu et al. review how nano-strategies can deliver CAF-modulating agents—such as cytotoxic drugs, signaling inhibitors, or phenotype-switching molecules—with enhanced specificity and reduced systemic toxicity. Combining CAF-targeted nanotherapies with ICIs has shown synergistic effects in preclinical models, revitalizing T cell infiltration and antitumor immunity.

In osteosarcoma, Fan et al. highlight CAF-driven resistance mechanisms, including exosome-mediated gene regulation, metabolic reprogramming, and inhibition of ferroptosis. They also discuss emerging CAF-directed therapies, such as TGF-β blockade, FAP depletion, and CAF-reprogramming approaches, which may be combined with conventional chemotherapy or immunotherapy to overcome resistance.

Despite these advances, several challenges still exist. CAF plasticity, functional heterogeneity, and the dual roles of certain CAF subsets—sometimes even exerting tumor-restraining effects—complicate therapeutic targeting. Moreover, the translational gap between preclinical experiments and human trials remains wide (4). As Wang et al. point out, future efforts should integrate single-cell sequencing and spatial multi-omics to decipher the spatiotemporal dynamics of CAF subpopulations and identify context-dependent vulnerabilities, and develop precision treatment strategies based on molecular subtyping to accelerate clinical application.

In conclusion, the collective evidence from these nine articles convincingly establishes CAFs as pivotal regulators of immune evasion and therapy resistance across multiple cancer types. Targeting CAFs—whether through depletion, reprogramming, or disruption of their signaling networks—holds great promise for enhancing the efficacy of existing therapies, particularly immunotherapies. However, achieving success will necessitate a thorough grasp of CAF biology, reliable biomarkers, and innovative combination strategies (5). As we move forward, a multidisciplinary approach that integrates stromal targeting with immune modulation and precision oncology will be essential to disrupt the resilient CAF-TME axis and improve outcomes for cancer patients.
